# Supplementary material for: Beta cell-derived cholecystokinin drives obesity-associated pancreatic adenocarcinoma development
Source: Nat Commun. 2026 Feb 27;17:3292. doi: 10.1038/s41467-026-69821-2 (PMC13066563; doi:10.1038/s41467-026-69821-2)
Supplement: Supplementary file 1 — Supplementary Information [file 41467_2026_69821_MOESM1_ESM.pdf]

SUPPLEMENTARY TABLE

Antibody Target	Species	Company	Product Number	Application	Dilution
Cholecystokinin	Rabbit	Immunostar	20078	IF/ICC/IHC/EM	1:100/ 1:100/ 1:1000/ 1:250
Insulin	Rat	R&D Systems	MAB1417	IF/ICC/EM	1:500/ 1:500/ 1:250
Glucagon	Rabbit	Sigma-Aldrich	SAB4501137	IF	1:400
Glucagon	Mouse	Sigma-Aldrich	G2654	iDISCO	1:2000
Ki67	Rabbit	Cell Signaling Technology	9129S	IF	1:100
Phospho-c-Jun (Ser73)	Rabbit	Cell Signaling Technology	3270S	IB	1:500
cJun	Rabbit	Cell Signaling Technology	9165S	IB	1:1000
Hsp90	Rabbit	Cell Signaling Technology	4877S	IB	1:5000
JUN/cJun	Rabbit	EpiCypher	13-2019	CUT&RUN	0.5 µg
H3K4me3	Rabbit	EpiCypher	13-0060	CUT&RUN	0.5 µg
IgG control	Rabbit	EpiCypher	13-0042	CUT&RUN	0.5 µg
Insulin	Guinea Pig	Accurate Chemical & Scientific Corporation	BMAT5014	IHC	1:300
BiP	Rabbit	Cell Signaling Technology	3177	IHC	1:400
Synaptophysin	Rabbit	ThermoFisher Scientific	RB-1461	IHC	1:100
Ki67	Rabbit	Biocare Medical	CRM325	IHC	1:75
Cd45	Rabbit	Abcam	ab10558	IHC	1:500
Smooth muscle actin (SMA)	Mouse	ThermoFisher Scientific	MS-113	IHC	1:400
Reg2	Goat	R&D Systems	AF2035	IHC	1:100
Anti-Rabbit IgG (DyLight 800 4X PEG conjugate)	Goat	Cell Signaling Technology	5151S	IB	1:2500
Anti-Rat IgG Alexa Fluor Plus 488	Donkey	ThermoFisher Scientific	A48269	IF/ICC	1:500
Anti-Rabbit IgG Alexa Fluor Plus 647	Donkey	ThermoFisher Scientific	A32795	IF/ICC	1:500
Mach2 Rabbit HRP-Polymer	Goat	Biocare Medical	RHRP520	IHC	1:1
Mach2 Mouse HRP-Polymer	Goat	Biocare Medical	MHRP520	IHC	1:1
Peroxidase AffiniPure Anti-Goat	Rabbit	Jackson ImmunoResearch	305-035-045	IHC	1:500
Peroxidase AffiniPure Anti-Guinea Pig	Donkey	Jackson ImmunoResearch	706-035-148	IHC	1:500
Biotinylated, Anti-Rabbit	Goat	Vector Laboratories	BA-1000	IHC	1:100

Supplementary Table 1. Antibodies used in the study. IF = immunofluorescence, ICC = immunocytochemistry, IHC = immunohistochemistry, EM = electron microscopy, IB = immunoblot

SUPPLEMENTARY FIGURES

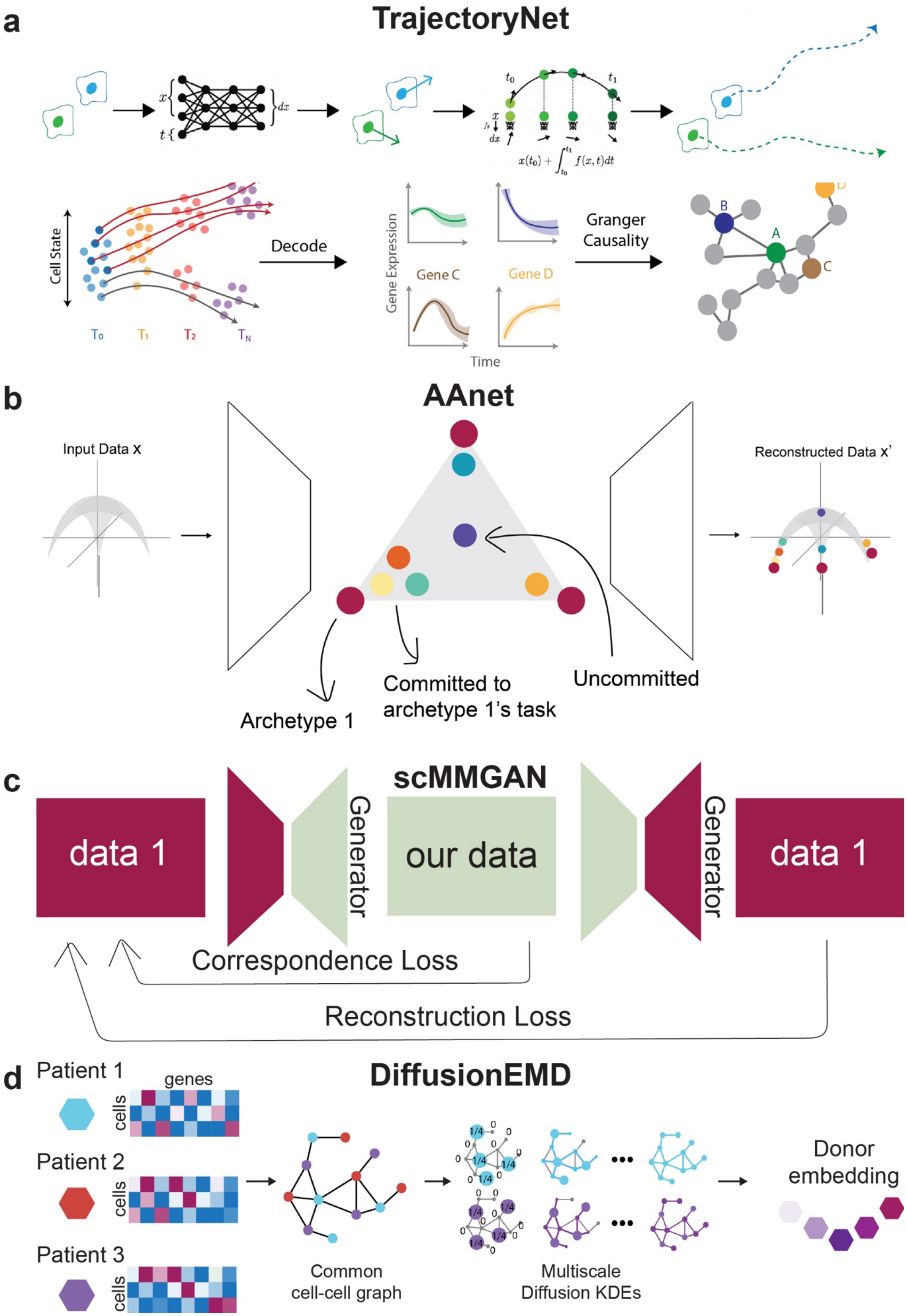

Supplementary Fig. 1. Overview of computational methods used in the study.
a TrajectoryNet learns cellular trajectories via a neural ODE-based optimal transport framework. With the *Cflows* framework, this enables transcriptional dynamics, cell-of-origin, and gene regulation analysis. **b** AAnet

transforms data into a latent representation shaped like a simplex for performing latent-space archetypal analysis. Combined with TrajectoryNet, AAnet characterizes archetypes and archetypes-of-origin, such as the cellular origin of *Cck*+ β cells. **c** scMMGAN is a generative adversarial network, which uses adversarial training to map published datasets onto new single-cell data. Combined with AAnet, scMMGAN identifies which archetypes are enriched in specific conditions or developmental stages, such as obesity or age. Combined with TrajectoryNet, scMMGAN assesses if the mapped cells show similar cellular dynamics. **d** DiffusionEMD builds an embedding of patient data based on similarity of cellular populations between patients, which enables characterization of a relationship between clinical variables of interest and cellular variation, such as that along the obesity progression axis.

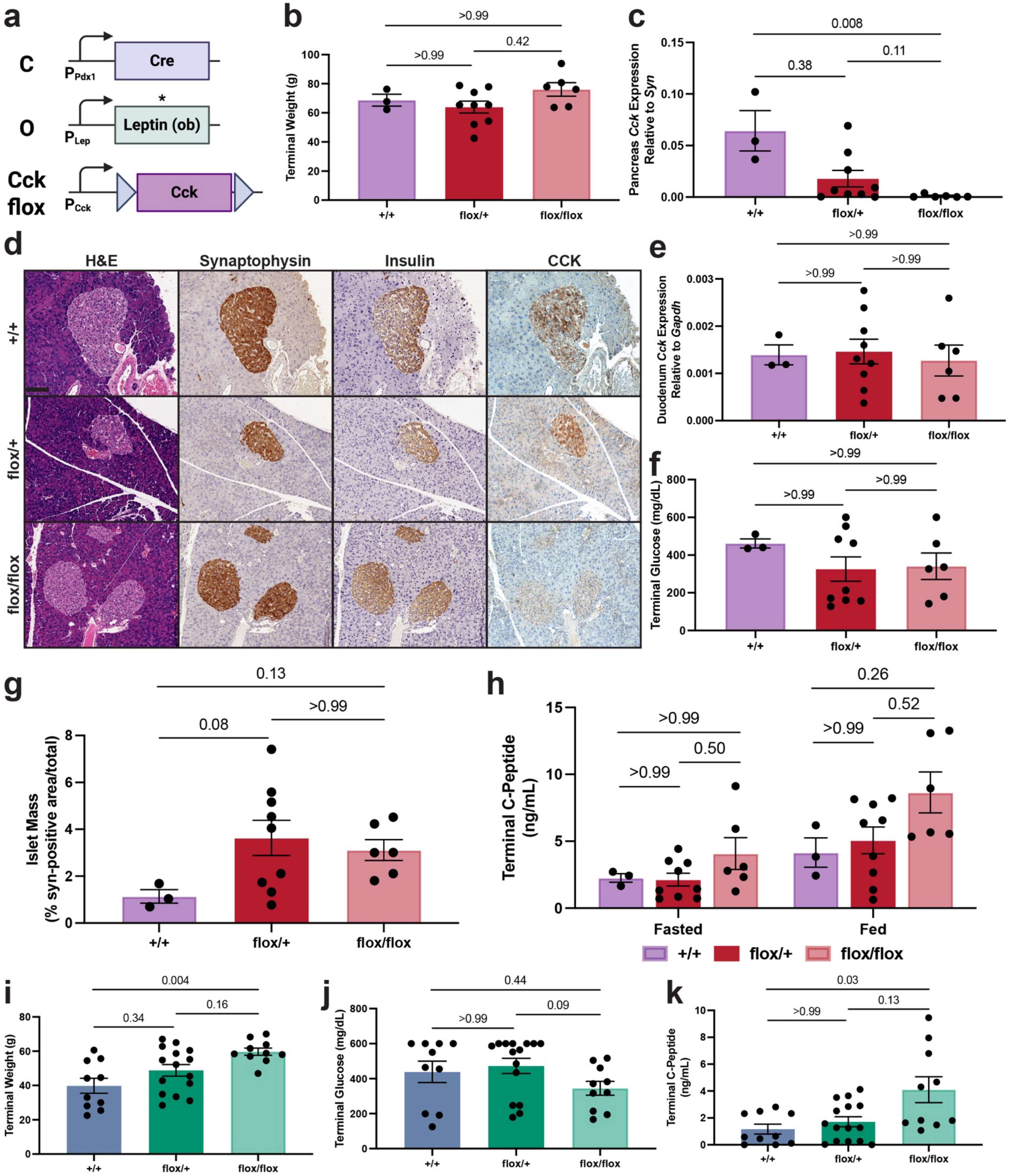

Supplementary Fig. 2. β cell CCK expression is dispensable for islet homeostasis in obese mice. **a** Schematic of alleles used to generate *Pdx1-Cre;Lep*$^{ob/ob}$, *Pdx1-Cre;Lep*$^{ob/ob}$*;Cck*$^{flox/+}$, and *Pdx1-Cre;Lep*$^{ob/ob}$*;Cck*$^{flox/flox}$ mice. Created in BioRender. McQuaid, D. (2026) https://BioRender.com/fqjwxwm. **b** Terminal weight (mean ± SEM) of *Pdx1-Cre;Lep*$^{ob/ob}$ mice of designated *Cck* genotypes. *p*-values of Kruskal-Wallis with Dunn's post-hoc test are shown. **c** Pancreatic *Cck* expression (qRT-PCR, mean ± SEM) normalized to synaptophysin (*Syn*) of *Pdx1-Cre;Lep*$^{ob/ob}$ mice of designated *Cck* genotypes. *p*-values of Kruskal-Wallis with

Dunn's post-hoc test are shown. **d** Images of CCK IHC on pancreata of *Pdx1-Cre;Lep*$^{ob/ob}$ mice of designated *Cck* genotypes. Images are representative of *n* = 3 mice per group. Scale bar, 100µm. **e** Duodenal *Cck* expression (qRT-PCR, mean ± SEM) normalized to *Gapdh* of *Pdx1-Cre;Lep*$^{ob/ob}$ mice of designated *Cck* genotypes. *p*-values of Kruskal-Wallis with Dunn's post-hoc test are shown. **f-h** Terminal random glucose (**f**), islet mass (**g**), and C-peptide under both 6-hour fasted and fed conditions (**h**) (mean ± SEM) of *Pdx1-Cre;Lep*$^{ob/ob}$ mice of designated *Cck* genotypes. *p*-values of Kruskal-Wallis with Dunn's post-hoc test are shown. For (**b-h**), 4-month-old *Pdx1-Cre;Lep*$^{ob/ob}$ (*n* = 3 male mice), *Pdx1-Cre;Lep*$^{ob/ob}$*;Cck*$^{flox/+}$ (*n* = 9 mice (3 male, 6 female)), and *Pdx1-Cre;Lep*$^{ob/ob}$*;Cck*$^{flox/flox}$ (*n* = 6 mice (3 male, 3 female)) littermates were analyzed. **i-k** Terminal weight (**i**), random glucose (**j**), and C-peptide (**k**) (mean ± SEM) of 3-month-old *KCO* (*n* = 10 mice (7 male, 3 female)), *KCO;Cck*$^{flox/+}$ (*n* = 15 mice (8 male, 7 female)), and *KCO;Cck*$^{flox/flox}$ (*n* = 10 mice (9 male, 1 female)) littermates. *p*-values of Kruskal-Wallis with Dunn's post-hoc test are shown. Source data are provided as a Source Data file.

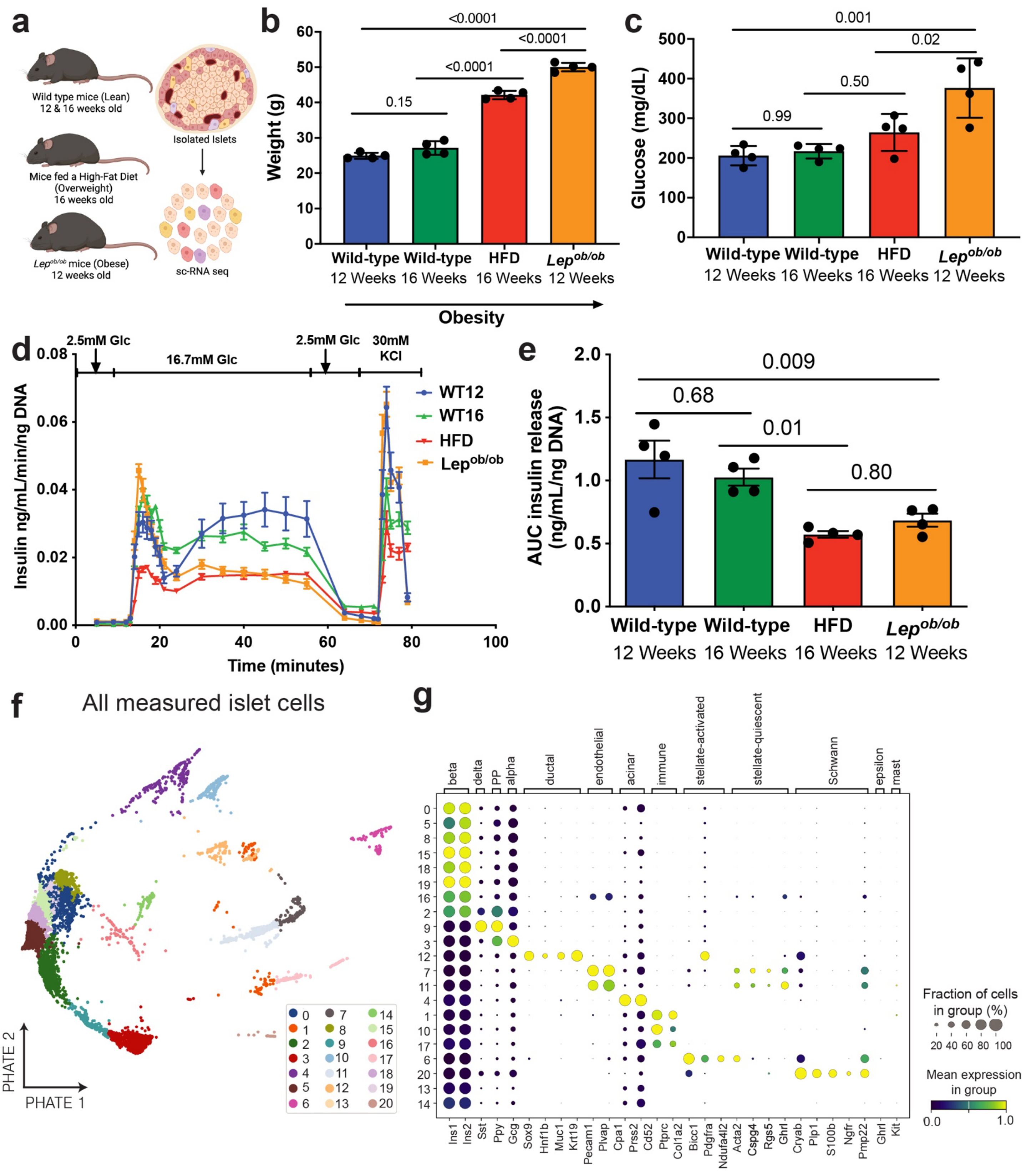

Supplementary Fig. 3. Single-cell RNA-sequencing of murine β cells from lean and obese models. **a** Overview of scRNA-seq experiment on mouse islets from congenic obesity models (high-fat diet (HFD)-fed for 10 weeks, *Lep*$^{ob/ob}$) and age-matched lean wild-type (WT) controls. Created in BioRender. Garcia, C. (2026) https://BioRender.com/kzxq91z. **b** Terminal weight (mean ± SEM) of mice in (**a**) (*n* = 4 male mice per group). Age listed refers to time of harvest. *p*-values for one-way ANOVA with Tukey's post-hoc test are shown. **c** Random glucose levels (mean ± SEM) of mice in (**a**) (*n* = 4 male mice per group). Age listed refers to time of harvest. *p*-values for one-way ANOVA with Tukey's post-hoc test are shown. **d** Glucose stimulated insulin secretion (GSIS) measurements (mean ± SEM, *n* = 4 biologic replicates per group) of islets isolated from

congenic WT (12 and 16 weeks of age (WT12, WT16), 16-week-old HFD-fed mice, and 12-week-old *Lep*$^{ob/ob}$ mice. Islets were pooled from *n* = 4 male mice per group but split into independent wells (n=4) that were independently stimulated and measured. Data from WT12 and *Lep*$^{ob/ob}$ were reanalyzed from our prior work[18]. **e** Area under the curve (AUC; mean ± SEM, *n* = 4 biologic replicates per group) for GSIS measurements in (**d**). *p*-values for one-way ANOVA with Tukey's post-hoc test are shown. **f** PHATE embedding of exocrine and endocrine islet cells (*n* = 23,469 cells), colored by cluster annotation. **g** Dot plot of marker gene expression (color scale represents min to max of mean normalized UMI, scaled per gene) for each cluster. Clusters 0, 5, 8, 15, 16, 18, and 19, were annotated as β cells; 2 as polyhormonal (insulin and another hormone); 9 as δ and PP cells; 3 as α cells; 12 as duct cells; 7 and 11 as endothelial cells; 4 as acinar cells; 1, 10, 17 as immune cells; 6 as stellate-activated cells; 20 as Schwann cells; and 13, 14 as low-quality clusters (see **Methods**). Source data are provided as a Source Data file.

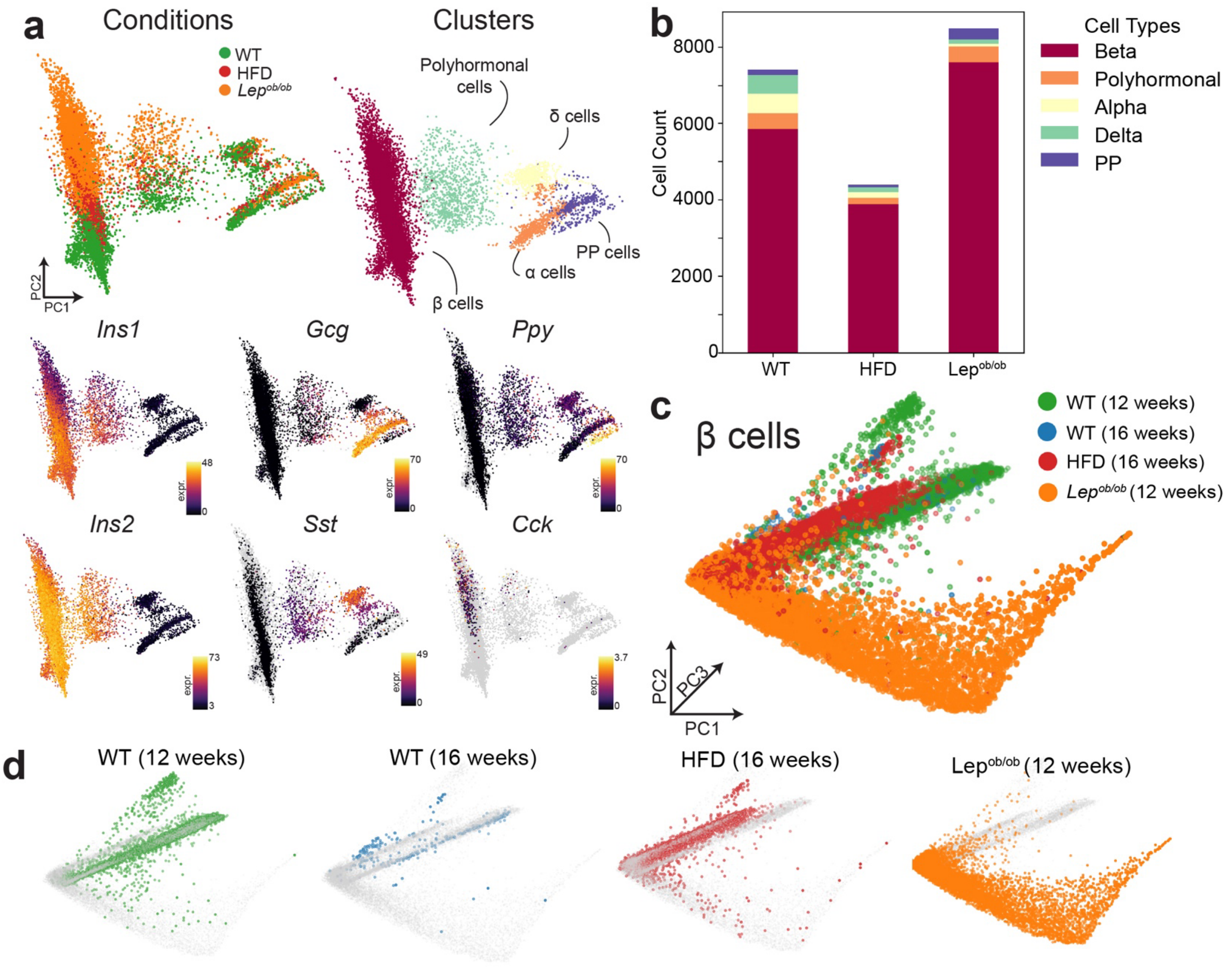

Supplementary Fig. 4. Single-cell RNA-sequencing reveals β cell adaptation to obesity.
a Combined embedding of endocrine cells (*n* = 20,294 cells) from three conditions (wild-type (WT), high-fat diet (HFD), and *Lep*$^{ob/ob}$) and annotated clusters based on marker gene expression (color scales represent min to max of normalized UMI, except *Cck* for which max is the 99th percentile; gray denotes 0 normalized UMI). **b** Endocrine cell type counts per condition. **c** Composite β cell embedding (*n* = 17,336 cells) colored by sample. **d** Composite β cell embedding (*n* = 17,336 cells) with individual samples plotted separately. Age of harvest is listed. Source data are provided as a Source Data file.

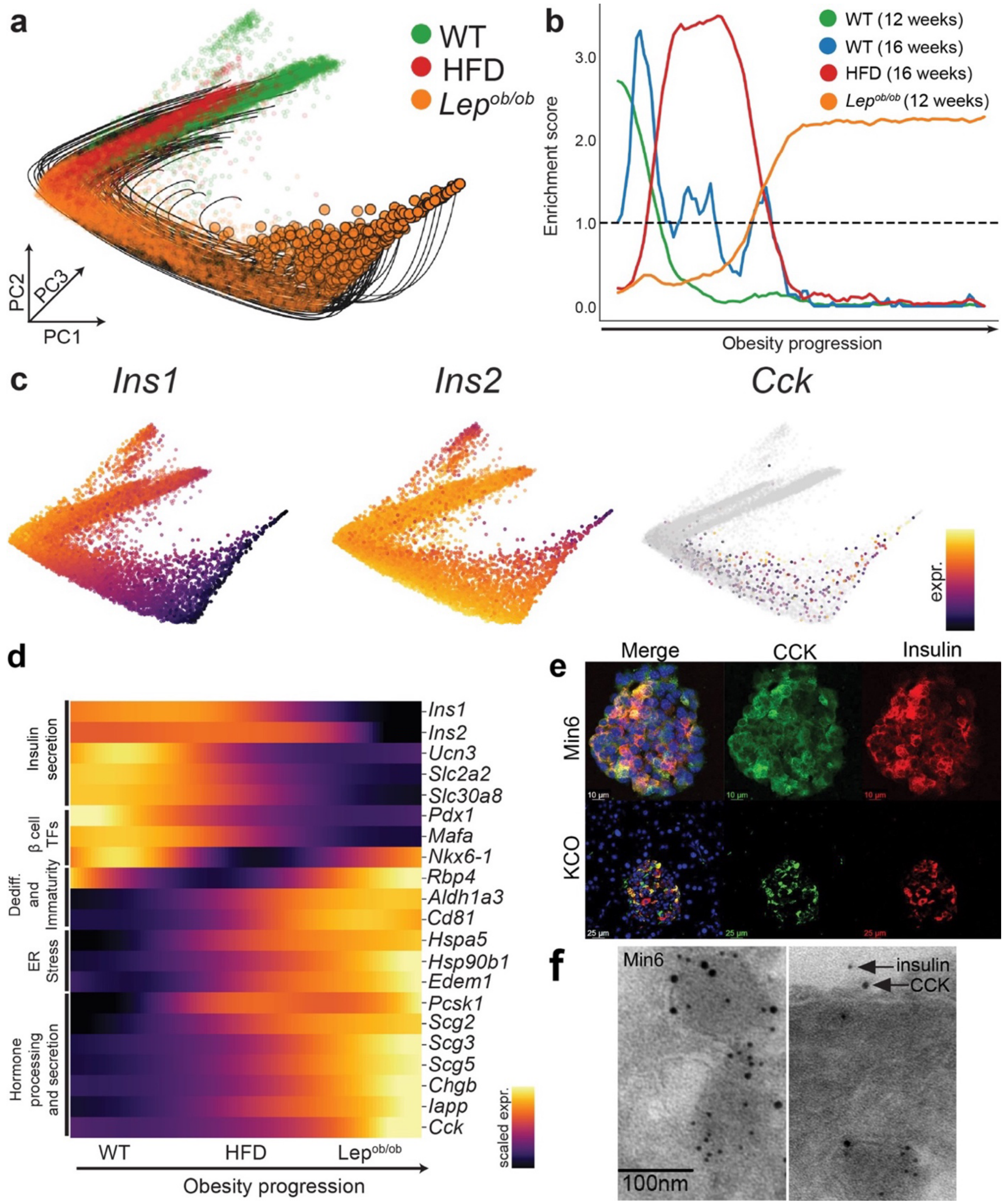

Supplementary Fig. 5. Dynamic changes in β cell hormone expression with obesity.
a β cell embedding (n = 17,336 cells) colored by condition (wild-type (WT), high-fat diet (HFD), and *Lep*$^{ob/ob}$). Visualized trajectories to high *Cck*-expressing cells on obesity progression axis are shown. **b** Enrichment scores (mean over n = 2 TrajectoryNet runs) for each sample within cells on the trajectory from WT to high *Cck*-expressing calculated at each timepoint, demonstrating trajectories are guided by obesity progression and not age-matched batch. **c** β cells (n = 17,336 cells) colored by insulin (*Ins1*, *Ins2*) and *Cck* scaled expression (color scale represents min to the 99th percentile of normalized UMI; gray denotes 0 normalized UMI). **d** Gene expression (color scale represents min to max of normalized UMI, scaled per gene) across all cells along the obesity progression axis in (**a**) for key marker genes. **e** Co-immunofluorescence images of mouse insulinoma cells (Min6) and *KCO* mice displaying nuclei (DAPI, blue), CCK (green), and insulin (red). **f** Co-immunoelectron microscopy images of Min6 cells labeled with insulin (5 nm dots) and CCK (10 nm dots). Arrows denote insulin and CCK at the plasma membrane, indicating possible active co-secretion. Scale bar, 100 nm. Source data are provided as a Source Data file.

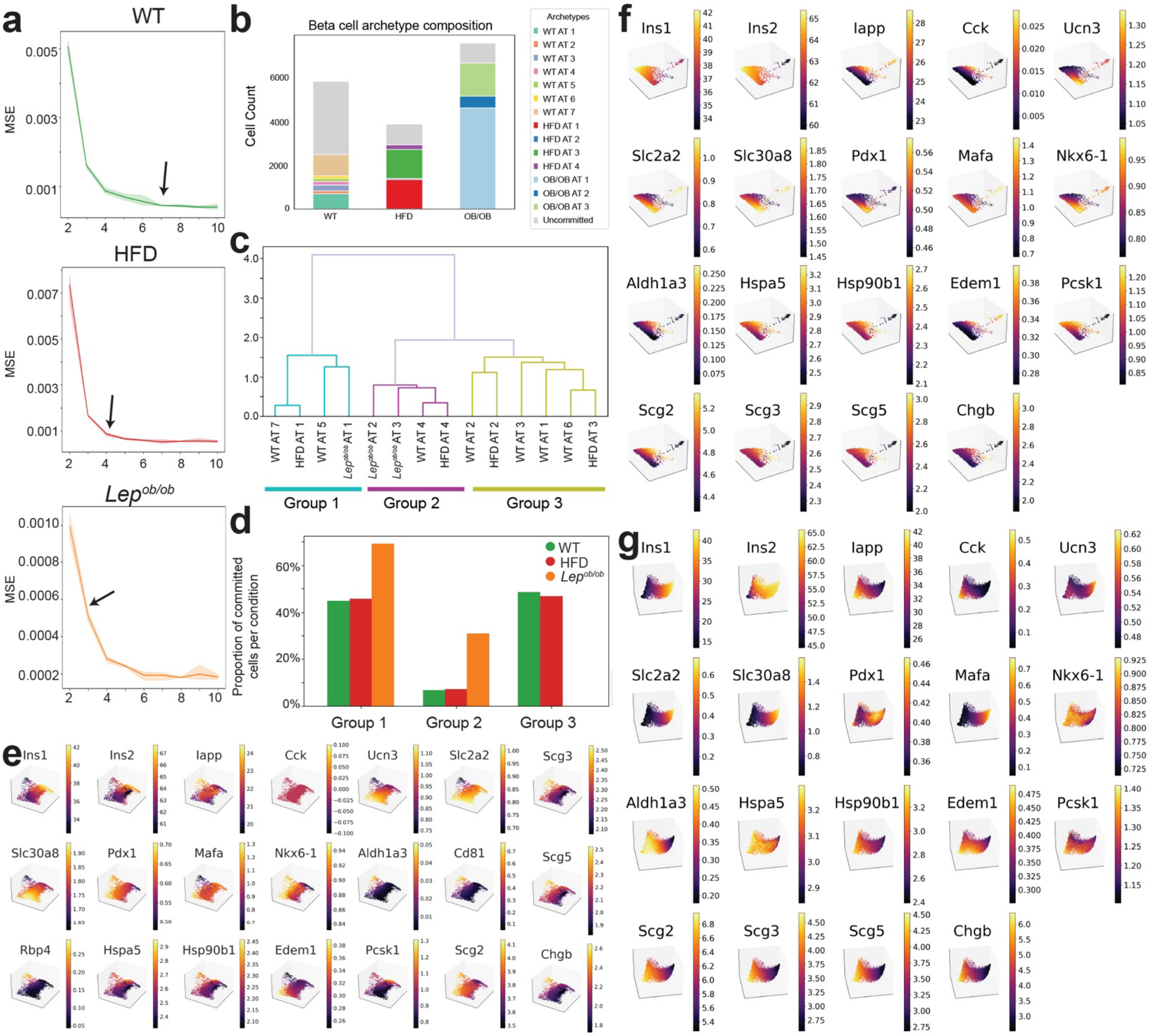

Supplementary Fig. 6. Analysis of β cell heterogeneity in mouse models of obesity.
a AAnet-inferred number of archetypes, computed based on elbow point of reconstruction error for held-out test set over number of archetypes from [2,10] (*n* = 3 runs per condition). **b** Count of cells for each condition and archetype. **c** Cosine similarity between archetypes defined across the entire transcriptome reveals three groups of archetypes shared across wild-type (WT), high-fat diet (HFD), and *Lep*$^{ob/ob}$ conditions. **d** Proportion of committed cells from each condition per archetypal group. **e** WT embedding (*n* = 5,847 cells) of archetypes colored by marker gene expression. **f** HFD embedding (*n* = 3,887 cells) of archetypes colored by marker gene expression (normalized UMI per cell). **g** *Lep*$^{ob/ob}$ embedding (*n* = 7,602 cells) of archetypes colored by marker gene expression (normalized UMI per cell). For (**e-g**), color scales represent min to max of denoised normalized UMI. Source data are provided as a Source Data file.

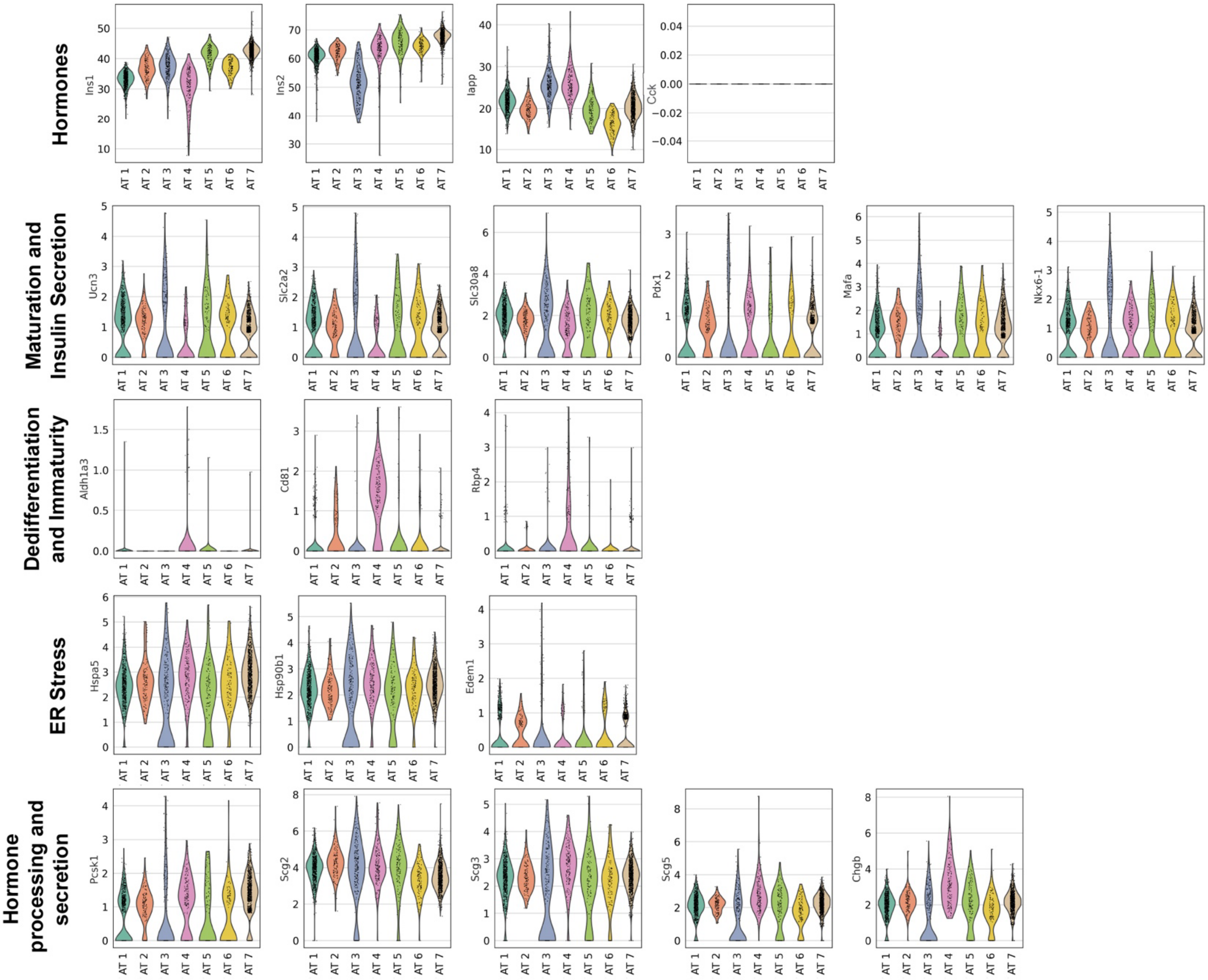

Supplementary Fig. 7. Marker gene expression and variability across wild-type (WT) archetypes (AT). Violin plots showing density estimates of the distribution of expression (normalized UMI per cell) and individual cells overlaid. WT AT 1 (*n* = 703 cells), WT AT 2 (*n* = 127 cells), WT AT 3 (*n* = 263 cells), WT AT 4 (*n* = 168 cells), WT AT 5 (*n* = 136 cells), WT AT 6 (*n* = 117 cells), WT AT 7 (*n* = 983 cells). *Cck* is not expressed in any AT. Source data are provided as a Source Data file.

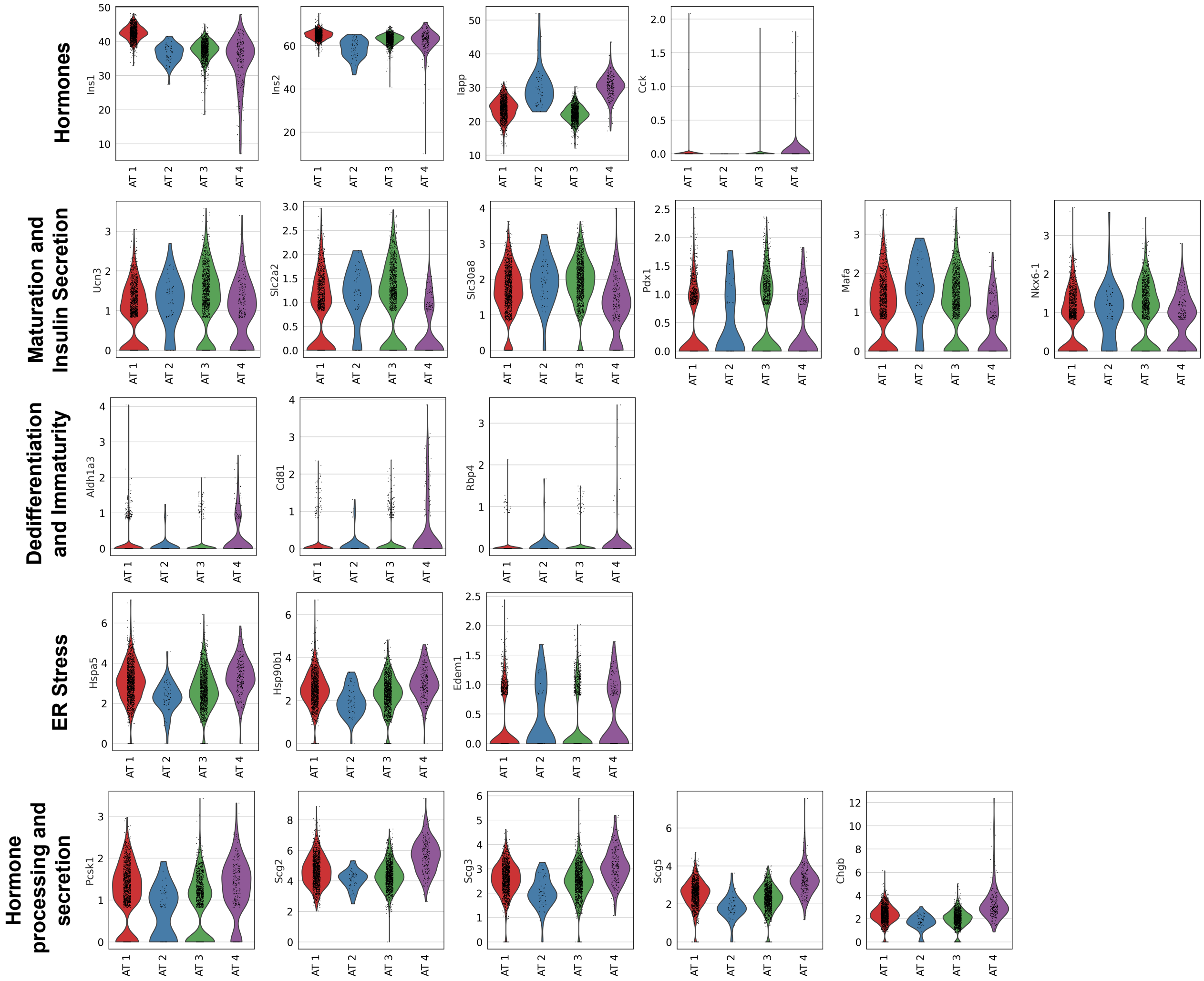

Supplementary Fig. 8. Marker gene expression and variability across high-fat diet (HFD) archetypes. Violin plots showing density estimates of the distribution of expression (normalized UMI per cell) and individual cells overlaid. HFD AT 1 (*n* = 1,347 cells), HFD AT 2 (*n* = 52 cells), HFD AT 3 (*n* = 1,329 cells), HFD AT 4 (*n* = 215 cells). Source data are provided as a Source Data file.

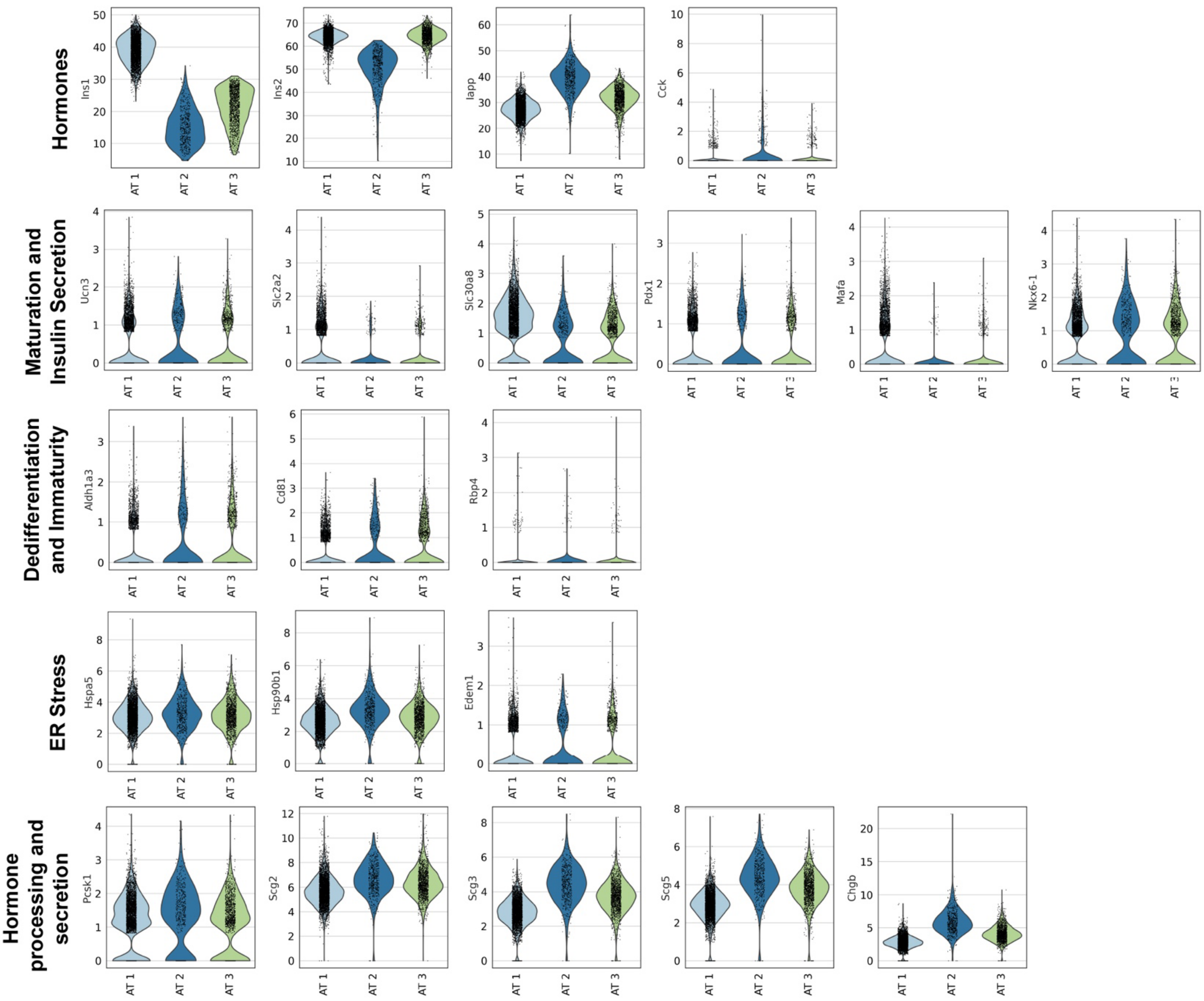

Supplementary Fig. 9. Marker gene expression and variability across *Lep*$^{ob/ob}$ archetypes.
Violin plots showing density estimates of the distribution of expression (normalized UMI per cell) and individual cells overlaid. *Lep*$^{ob/ob}$ AT 1 (*n* = 4,628 cells), *Lep*$^{ob/ob}$ AT 2 (*n* = 546 cells), *Lep*$^{ob/ob}$ AT 3 (*n* = 1,522 cells). Source data are provided as a Source Data file.

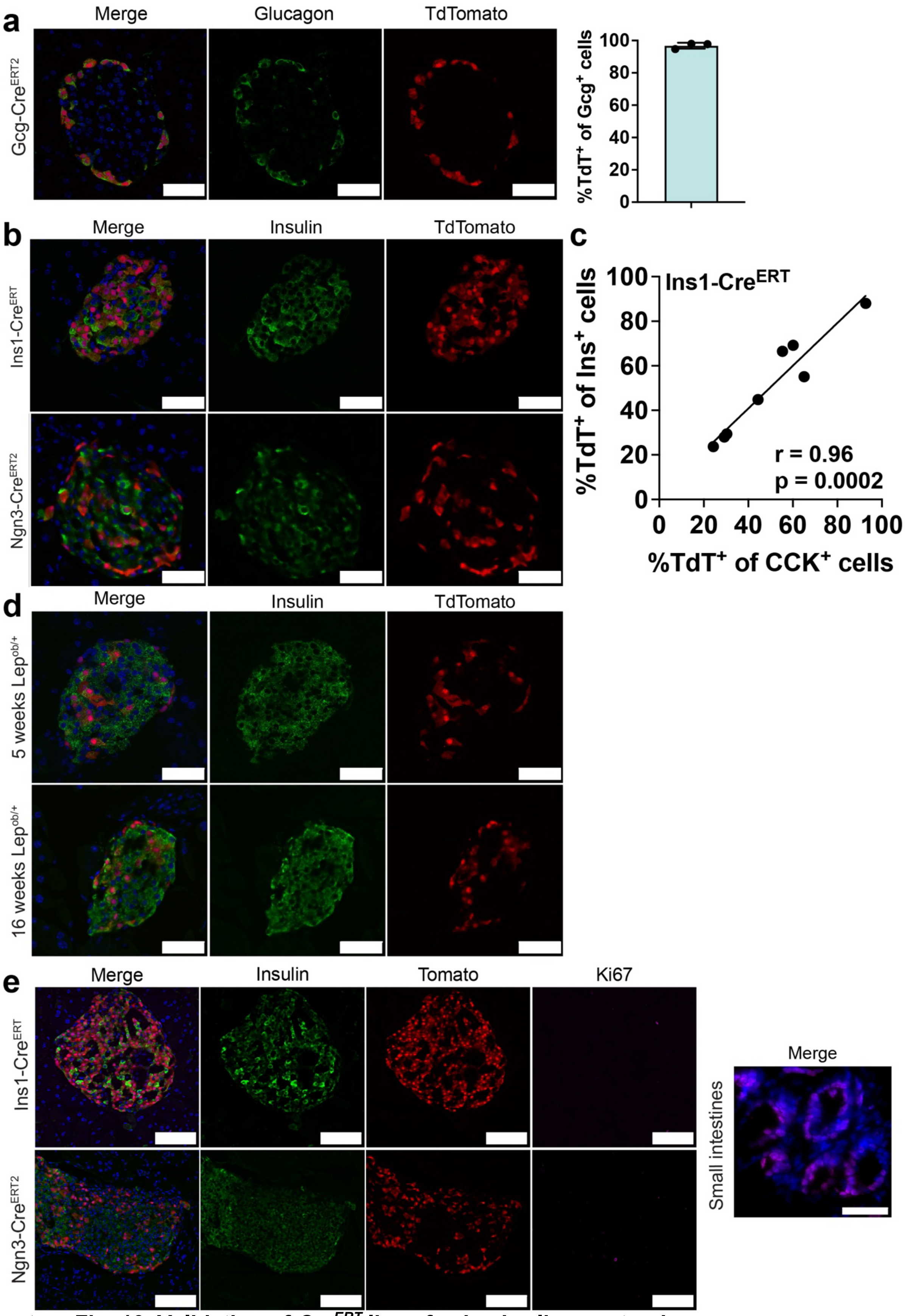

Supplementary Fig. 10. Validation of *Cre^{ERT}* lines for *in vivo* lineage tracing.

a Images of Gcg immunofluorescence and lineage-traced TdTomato+ cells in 5-week-old *Gcg-Cre^{ERT2};Lep$^{ob/ob}$;Rosa26$^{LSL\text{-}TdTomato}$* mice administered tamoxifen at 4 weeks of age. DAPI labels nuclei blue. Scale bar, 50μm. Average percentage (mean ± SEM, *n* = 3 mice (2 male, 1 female)) of Gcg+ cells labeled with TdTomato in littermates is shown showing near complete overlap. **b** Images of insulin immunofluorescence

and lineage-traced TdTomato+ cells in 5-week-old *Ins1-Cre*^{ERT}*;Lep*$^{ob/ob}$*;Rosa26*$^{LSL\text{-}TdTomato}$ (representative of *n* = 4 mice) and *Ngn3-Cre*^{ERT2}*;Lep*$^{ob/ob}$*;Rosa26*$^{LSL\text{-}TdTomato}$ (representative of *n* = 2 mice) mice administered tamoxifen at 4 weeks of age. DAPI labels nuclei blue. Scale bar, 50µm. **c** Strong positive correlation (Pearson correlation analysis, *r* = 0.96, *p* = 0.0002) between total labeled insulin+/TdTomato+ and CCK+/TdTomato+ cells in 16-week-old *Ins1-Cre*^{ERT}*;Lep*$^{ob/ob}$*;Rosa26*$^{LSL\text{-}TdTomato}$ littermates administered tamoxifen at 4 weeks of age (*n* = 8 mice (4 male, 4 female). **d** Images of insulin immunofluorescence and lineage-traced TdTomato+ cells in 5-week-old (representative of *n* = 2 mice) and 16-week-old (representative of *n* = 4 mice) lean *Ngn3-Cre*^{ERT2}*;Lep*$^{ob/+}$*;Rosa26*$^{LSL\text{-}TdTomato}$ mice administered tamoxifen at 4 weeks of age. DAPI labels nuclei blue. Scale bar, 50µm. **e** Co-immunofluorescence for insulin and Ki67 of islets from 16-week-old *Ins1-Cre*^{ERT}*;Lep*$^{ob/ob}$*;Rosa26*$^{LSL\text{-}TdTomato}$ and *Ngn3-Cre*^{ERT2}*;Lep*$^{ob/ob}$*;Rosa26*$^{LSL\text{-}TdTomato}$ mice shows no Ki67+ cells within the islet (DAPI: blue, insulin: green, TdTomato: red, Ki67: magenta). Scale bar, 100µm. Small intestine from 16-week-old *Ins1-Cre*^{ERT}*;Lep*$^{ob/ob}$*;Rosa26*$^{LSL\text{-}TdTomato}$ mice demonstrates Ki67+ cells in the crypts as a positive control. Scale bar, 50µm. DAPI labels nuclei blue. Source data are provided as a Source Data file.

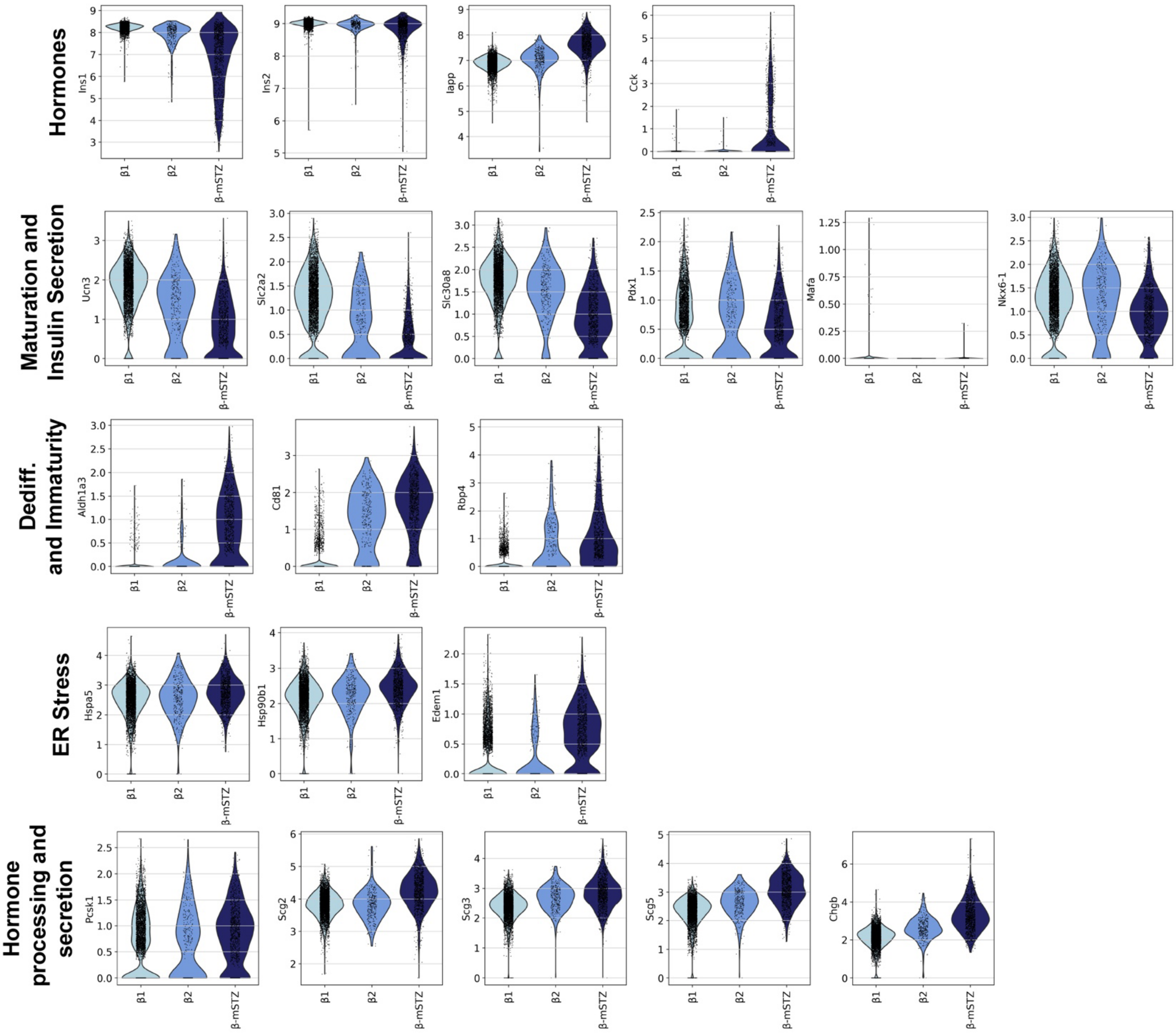

Supplementary Fig. 11. Marker gene expression and variability across STZ and control β cell subclusters.

Violin plots depicting density estimates of the distribution of expression (normalized UMI per cell) with individual cells overlaid belonging to STZ and control β cell subclusters. β1 (*n* = 5,332 cells), β2 (*n* = 304 cells), β-mSTZ (*n* = 1,124 cells). Source data are provided as a Source Data file.

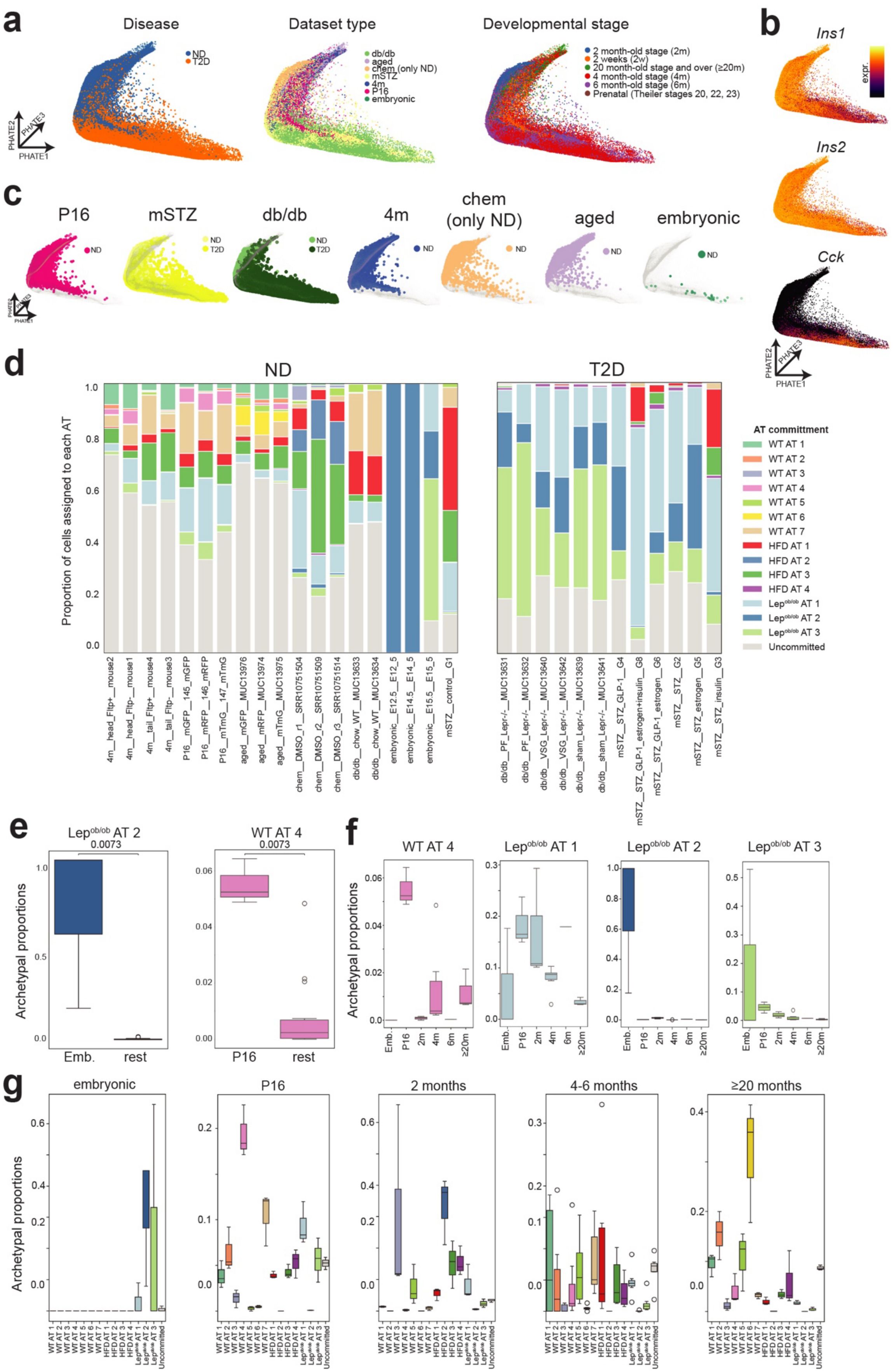

Supplementary Fig. 12. Comparison of obesity progression to β cells from non-diabetic (ND) and type II diabetes (T2D) mouse models.

a Embedding of mouse atlas β cells (*n* = 85,129 cells) mapped onto the obesity progression with scMMGAN colored by disease annotation (ND: non-diabetic, T2D: Type II diabetes), dataset type, and development stage.

b Insulin (*Ins1*, *Ins2*) and *Cck* scaled gene expression (color scale represents min to max of normalized UMI) in the mouse atlas β cells mapped onto the obesity progression. **c** Embedding with each mapped mouse dataset plotted separately. P16 (n = 7,410 cells); mSTZ (ND n = 5,795 cells, T2D n = 8,534 cells); db/db (ND n = 7,706 cells, T2D n = 24,277); 4m (n = 13,738 cells). chem (n = 3,319 cells); aged (n = 14,317 cells); embryonic (n = 33 cells). **d** Proportion of each archetype for each ND and T2D dataset. **e** Archetypal proportion for Embryonic (n = 3 samples) versus rest of ND (n = 16 samples) and P16 (n = 3 samples) versus rest of ND (n = 16 samples). Box plots display 25th, 50th, and 75th percentiles ± 1.5 interquartile range (IQR). *p*-values of two-sided Wilcoxon rank sum test are shown. **f** Archetypal proportion for WT AT 4, *Lep^ob/ob^* AT 1, *Lep^ob/ob^* AT 2, and *Lep^ob/ob^* AT 3 for ND datasets ordered by age (embryonic (n = 3 samples), P16 (n = 3 samples), 2m (n = 3 samples), 4m (n = 6 samples), 6m (n = 1 sample), aged (≥20 months, n = 3 samples)). Box plots display 25th, 50th, and 75th percentiles ± 1.5 interquartile range (IQR). **g** Archetypal proportion for ND datasets from each age (embryonic (n = 3 samples), P16 (n = 3 samples), 2m (n = 3 samples), 4m (n = 6 samples), 6m (n = 1 sample), aged (≥20 months, n = 3 samples)). Box plots display 25th, 50th, and 75th percentiles ± 1.5 interquartile range (IQR). Source data are provided as a Source Data file.

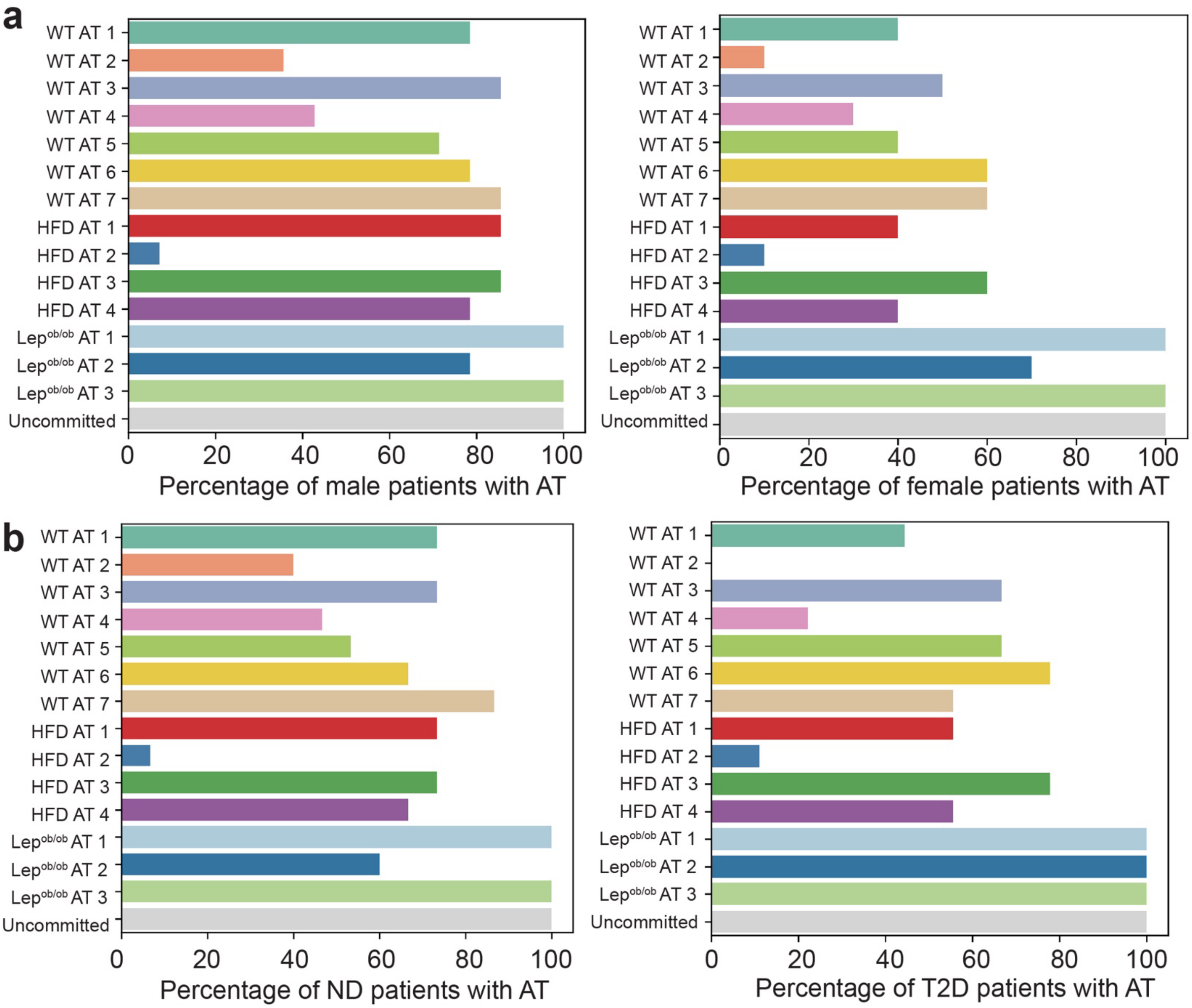

Supplementary Fig. 13. Percentage of patients harboring cells assigned to each archetype.
a Percentage of male and female patients harboring each archetype (AT). **b** Percentage of ND (non-diabetic) and T2D (type II diabetic) patients harboring each AT. Source data are provided as a Source Data file.

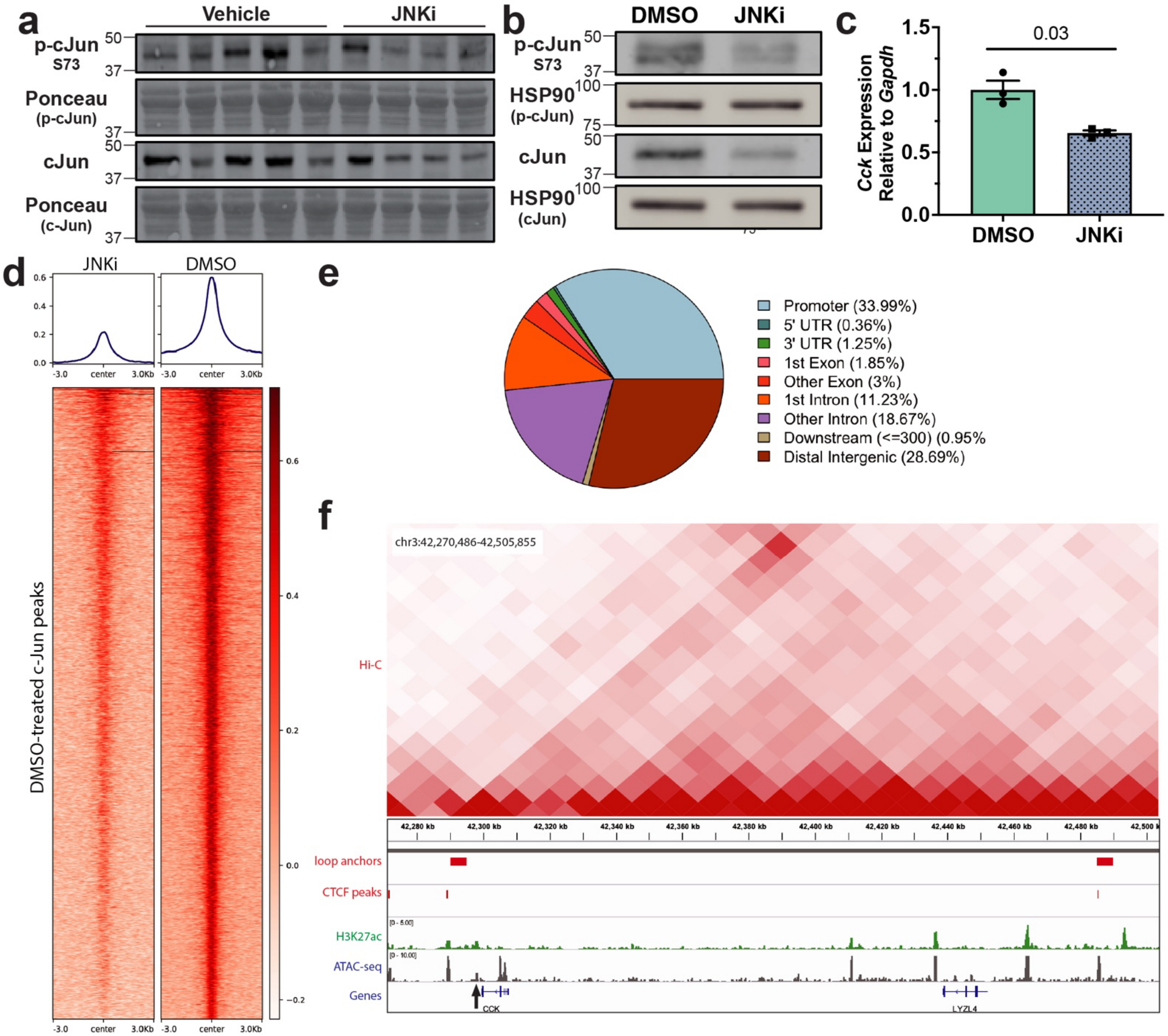

Supplementary Fig. 14. JNK/cJun signaling regulates β cell CCK expression.
a Western blot of total cJun and phospho-cJun S73 of pancreatic lysates from 16-week-old *Lep*$^{ob/ob}$ mice treated with JNKi (*n* = 4 mice; 20 mg/kg SP600125) or vehicle (*n* = 5 mice) for 5 days. Ponceau is protein loading control with associated primary antibody listed in parenthesis. The samples derived from the same experiment but were processed in parallel in different gels for cJun and p-cJun. **b** Western blot (representative of *n* = 3 biologic replicates) of total cJun and phospho-cJun S73 in Min6 cells treated with JNK inhibitor (JNKi, 20 µM SP600125) or control (DMSO) for 48 hours. HSP90 is loading control with associated primary antibody listed in parenthesis. The samples derived from the same experiment but were processed in parallel in different gels for cJun and p-cJun. **c** Relative *Cck* gene expression (qRT-PCR, mean ± SEM, *n* = 3 biologic replicates) normalized to *Gapdh* of Min6 cells in (**b**). *p*-value of two-sided Welch's *t*-test is shown. **d** Aggregation heatmap plots of cJun CUT&RUN in Min6 cells treated with JNKi or DMSO control (average of *n* = 2 biologic replicates per group). cJun signal is normalized to E. coli spike-in and IgG. cJun signal is centered on cJun peaks from DMSO-treated cells. **e** Proportion of cJun binding in annotated genomic regions. **f** H3K27ac ChIP-seq and ATAC-seq show a conserved putative enhancer downstream of *CCK* in human islets (arrow). Hi-C demonstrates that the *CCK* putative enhancer occurs at the boundary of a chromatin loop denoted by loop anchors and CTCF bindings sites. Source data are provided as a Source Data file.

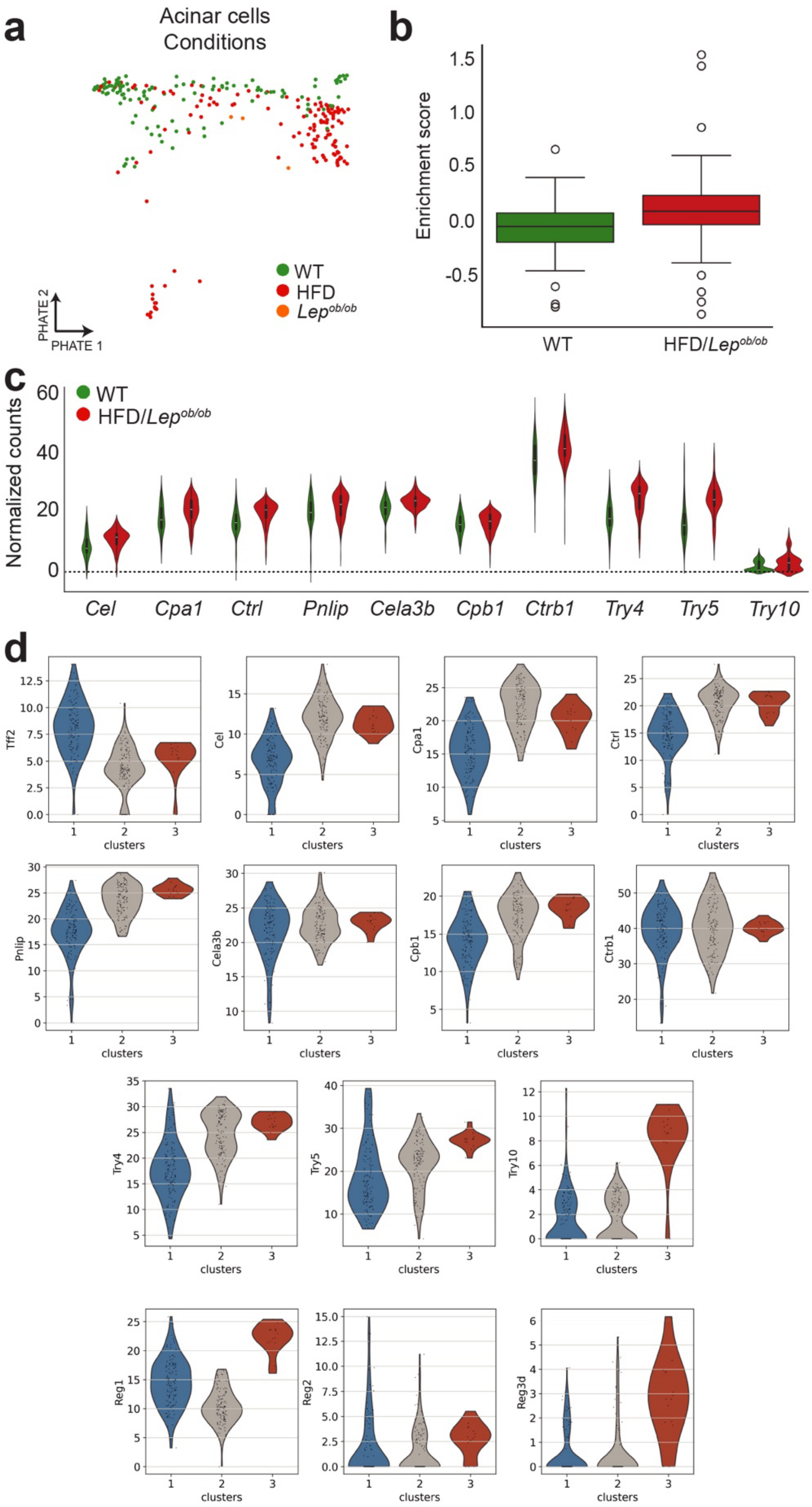

Supplementary Fig. 15. Marker gene expression and variability across acinar cells.
a PHATE visualization of acinar cells (*Cpa1+/Prss2+, n* = 260 cells) colored by sample condition. **b** Enrichment of gene signatures observed in peri-islet acinar cells from *Lepr*$^{db/db}$ mice in lean (WT *n* = 117 cells) and obese (HFD/*Lep*$^{ob/ob}$ *n* = 143 cells) models. Box plots display 25th, 50th, and 75th percentile enrichment scores ± 1.5 interquartile range (IQR) for each cell in each condition. **c** Violin plots represent protease expression distribution (min/max with 25th, 50th, and 75th percentiles delineated by lines) in acinar cells. Cell numbers same as (**b**). **d** Violin plots depicting density estimates of the distribution of expression (normalized UMI per cell) with individual cells overlaid belonging to each acinar subcluster (Cluster 1 *n* = 121 cells, Cluster 2 *n* = 124 cells, Cluster 3 *n* = 15 cells). Source data are provided as a Source Data file.
