# Supplementary material for: Beta cell-derived cholecystokinin drives obesity-associated pancreatic adenocarcinoma development
Source: Nat Commun. 2026 Feb 27;17:3292. doi: 10.1038/s41467-026-69821-2 (PMC13066563; doi:10.1038/s41467-026-69821-2)
Supplement: Supplementary file 2 — Descriptions of Additional Supplementary Files [file 41467_2026_69821_MOESM2_ESM.docx]

**Descriptions of Additional Supplementary Files**

**SUPPLEMENTARY DATA**

**Supplementary Data 1.** Two-sided Wilcoxon rank sum test results for all genes (log fold-change and Benjamini-Hochberg (BH)-adjusted *p*-value) for each archetype versus rest within each condition (WT, HFD, and *Lep^ob/ob^* (ob.ob)).

**Supplementary Data 2.** Enriched (*q* < 0.05) genes from gene set enrichment analysis with BioPlanet, KEGG, Reactome, and GO Biological Process for genes that (a) decrease and (b) increase in expression along the obesity progression axis. Gene set over-representation analysis was performed using Enrichr with Fisher’s exact (hypergeometric) test and Benjamini-Hochberg FDR correction, using default gene background.

**Supplementary Data 3.** Gene sets and associated genes enriched (*q* < 0.05) with respect to each human stressor and genes increasing over the obesity progression axis. Gene set over-representation analysis was performed using Enrichr with Fisher’s exact (hypergeometric) test and Benjamini-Hochberg FDR correction, using default gene background.

**Supplementary Data 4.** scMMGAN evaluation of mapping with batch ASW and global distortion (cosine similarity before and after alignment) for (**a**) vehicle cells (from mSTZ experiment) to WT cells, (**b**) mSTZ-treated cells to *Lep^ob/ob^* cells, (**c**) mouse atlas cells to WT, HFD, and *Lep^ob/ob^* cells, and (**d**) human ND and T2D donor cells to WT, HFD, and *Lep^ob/ob^* cells.

**Supplementary Data 5.** Gene regulatory network, with subnetwork of genes identified as increasing over the obesity progression axis annotated and visualized in **Fig. 7b**.

**Supplementary Data 6.** Enriched (*q* < 0.05) genes from gene set enrichment analysis with BioPlanet, KEGG, Reactome, and GO Biological Process for subnetwork of genes identified as increasing over the obesity progression axis. Gene set over-representation analysis was performed using Enrichr with Fisher’s exact (hypergeometric) test and Benjamini-Hochberg FDR correction, using default gene background.

**SUPPLEMENTARY MOVIES**

**Supplementary Movie 1.** 3D reconstruction of tumor-bearing pancreas from *KC;Lep^ob/+^* mouse following tissue clearing and light sheet microscopy. Disease lesions are depicted in gray with islets colored by proximity.

**Supplementary Movie 2.** 3D reconstruction of tumor-bearing pancreas from *KC;Lep^ob/ob^* mice following tissue clearing and light sheet microscopy. Disease lesions are depicted in in gray with islets colored by proximity.
