# Supplementary material for: Beta cell-derived cholecystokinin drives obesity-associated pancreatic adenocarcinoma development
Source: Nat Commun. 2026 Feb 27;17:3292. doi: 10.1038/s41467-026-69821-2 (PMC13066563; doi:10.1038/s41467-026-69821-2)
Supplement: Supplementary file 11 — Reporting Summary [file 41467_2026_69821_MOESM11_ESM.pdf]

nature portfolio

Corresponding author(s): Mandar Deepak Muzumdar, Smita Krishnaswamy

Last updated by author(s): 1/23/2026

Reporting Summary

Nature Portfolio wishes to improve the reproducibility of the work that we publish. This form provides structure for consistency and transparency in reporting. For further information on Nature Portfolio policies, see our Editorial Policies and the Editorial Policy Checklist.

Statistics

For all statistical analyses, confirm that the following items are present in the figure legend, table legend, main text, or Methods section.

n/a	Confirmed	
☐	☒	The exact sample size (*n*) for each experimental group/condition, given as a discrete number and unit of measurement
☐	☒	A statement on whether measurements were taken from distinct samples or whether the same sample was measured repeatedly
☐	☒	The statistical test(s) used AND whether they are one- or two-sided<br>*Only common tests should be described solely by name; describe more complex techniques in the Methods section.*
☐	☒	A description of all covariates tested
☐	☒	A description of any assumptions or corrections, such as tests of normality and adjustment for multiple comparisons
☐	☒	A full description of the statistical parameters including central tendency (e.g. means) or other basic estimates (e.g. regression coefficient) AND variation (e.g. standard deviation) or associated estimates of uncertainty (e.g. confidence intervals)
☐	☒	For null hypothesis testing, the test statistic (e.g. *F*, *t*, *r*) with confidence intervals, effect sizes, degrees of freedom and *P* value noted<br>*Give P values as exact values whenever suitable.*
☒	☐	For Bayesian analysis, information on the choice of priors and Markov chain Monte Carlo settings
☒	☐	For hierarchical and complex designs, identification of the appropriate level for tests and full reporting of outcomes
☐	☒	Estimates of effect sizes (e.g. Cohen's *d*, Pearson's *r*), indicating how they were calculated

Our web collection on statistics for biologists contains articles on many of the points above.

Software and code

Policy information about availability of computer code

Data collection	All software used in data collection is listed in Methods.
Data analysis	Statistical comparisons of mouse data were performed using Prism v10. All single-cell RNA-sequencing data were processed with Cell Ranger (v3.0.2). Code for TrajectoryNet v0.2.4, DiffusionEMD v0.5.0, MAGIC v3.0.0, PHATE v1.0.11, AANet v0.0, and scMMGAN v0.0 used in single-cell RNA-sequencing analyses has been deposited in the Krishnaswamy lab repository in Github: https://github.com/KrishnaswamyLab/Beta-Cell-Driven-PDAC. Highly variable genes for gene cluster analysis, GSEA of gene clusters, and Granger causality analysis were defined using default parameters in Scanpy v.1.9.3. Default hyperparameters were used for all analyses and associated analyzed data for this mansucript can be found at https://github.com/KrishnaswamyLab/Beta-Cell-Driven-PDAC. Bulk RNA-seq analyses were performed using Trim Galore v0.6.7, CutAdapt v3.5, STAR aligner v2.7.9, Cand DESeq2. CUT&RUN sequencing data were analyzed with Trimmomatic, FastQC, IGV, Bowtie2 v2.4.2, SAMTools v1.16, Macs2 v2.2.7.1, Deeptools v3.5.1, BEDTools v2.30, and MEME v5.4.1.

For manuscripts utilizing custom algorithms or software that are central to the research but not yet described in published literature, software must be made available to editors and reviewers. We strongly encourage code deposition in a community repository (e.g. GitHub). See the Nature Portfolio guidelines for submitting code & software for further information.

Data

Policy information about availability of data

All manuscripts must include a data availability statement. This statement should provide the following information, where applicable:

- Accession codes, unique identifiers, or web links for publicly available datasets
- A description of any restrictions on data availability
- For clinical datasets or third party data, please ensure that the statement adheres to our policy

New scRNA-seq, bulk RNA-seq, and CUT&RUN sequencing data generated in this study were deposited into the Gene Expression Omnibus (GEO) under accession numbers GSE279485 (https://www.ncbi.nlm.nih.gov/geo/query/acc.cgi?acc=GSE279485), GSE287290 (https://www.ncbi.nlm.nih.gov/geo/query/acc.cgi?acc=GSE287290), and GSE280947 (https://www.ncbi.nlm.nih.gov/geo/query/acc.cgi?acc=GSE280947), respectively. Accession numbers for reanalyzed data are listed above. All other data are included in the main text, supplementary information, or source data file. Source data are provided with this paper. There are no restrictions on data availability. Our previously published mouse scRNA-seq data18 were obtained from GEO (GSE137236: https://www.ncbi.nlm.nih.gov/geo/query/acc.cgi?acc=GSE137236). The following published datasets from ENCODE66,67 were used: H3K27ac ChIP-seq from mouse cortex (ENCSR491RBV: https://www.encodeproject.org/experiments/ENCSR491RBV/); H3K27ac ChIP-seq from mouse hippocampus (ENCSR527FRO: https://www.encodeproject.org/experiments/ENCSR527FRO/); DNase-seq from mouse hippocampus (ENCSR544UYQ: https://www.encodeproject.org/experiments/ENCSR544UYQ/); and CTCF ChIP-seq from human adult pancreas tissue (ENCSR774PGN: https://www.encodeproject.org/experiments/ENCSR774PGN/). H3K27ac ChIP-seq and ATAC-seq from human islets were obtained from Miguel-Escalada et al.68 (https://ega-archive.org/studies/EGAS00001002917). Loop anchor and Hi-C data (https://cmdga.org/experiments/TSTSR043623/) from human islets were obtained from Greenwald et al.69. The following human islet scRNA-seq datasets were accessed from GEO: GSE101207 (https://www.ncbi.nlm.nih.gov/geo/query/acc.cgi?acc=GSE101207), GSE81608 (https://www.ncbi.nlm.nih.gov/geo/query/acc.cgi?acc=GSE81608), GSE86469 (https://www.ncbi.nlm.nih.gov/geo/query/acc.cgi?acc=GSE86469), GSE154126 (https://www.ncbi.nlm.nih.gov/geo/query/acc.cgi?acc=GSE154126), GSE83139 (https://www.ncbi.nlm.nih.gov/geo/query/acc.cgi?acc=GSE83139), and GSE237448 (https://www.ncbi.nlm.nih.gov/geo/query/acc.cgi?acc=GSE237448). Published mouse pancreatic islet scRNA-seq data were accessed from (GSE211799: https://cellxgene.cziscience.com/collections/296237e2-393d-4e31-b590-b03f74ac5070) and GSE211799 (https://www.ncbi.nlm.nih.gov/geo/query/acc.cgi?acc=GSE211799).

Research involving human participants, their data, or biological material

Policy information about studies with human participants or human data. See also policy information about sex, gender (identity/presentation), and sexual orientation and race, ethnicity and racism.

Reporting on sex and gender	Sex data were extracted for patients included in human scRNA-seq data reanalyses of previously published datasets (GSE101207, GSE81608, GSE86469, GSE154126, and GSE83139) and were used as a variable in DiffusionEMD-based analyses for correlation to the obesity trajectory.
Reporting on race, ethnicity, or other socially relevant groupings	Race, ethnicity, and other social relevant groupings were not available in published datasets for inclusion in reanalyses.
Population characteristics	Population characteristics of reanalyzed human scRNA-seq data are available in the GEO submission (GSE101207, GSE81608, GSE86469, GSE154126, and GSE83139) and/or relevant publications.
Recruitment	Recruitment methods for patients included in human scRNA-seq data reanalyses are available in the GEO submission (GSE101207, GSE81608, GSE86469, GSE154126, and GSE83139) and/or relevant publications.
Ethics oversight	Ethics oversight for human scRNA-seq data collection used in reanalyses are available in the GEO submission (GSE101207, GSE81608, GSE86469, GSE154126, and GSE83139) and/or relevant publications.

Note that full information on the approval of the study protocol must also be provided in the manuscript.

Field-specific reporting

Please select the one below that is the best fit for your research. If you are not sure, read the appropriate sections before making your selection.

☒ Life sciences ☐ Behavioural & social sciences ☐ Ecological, evolutionary & environmental sciences

For a reference copy of the document with all sections, see nature.com/documents/nr-reporting-summary-flat.pdf

Life sciences study design

All studies must disclose on these points even when the disclosure is negative.

Sample size	For all experiments, no predetermined power calculations were performed for sample size calculation, as expected central tendencies and variances were not known in advance for most conditions. Sample sizes used for mouse, single-cell analyses, and in vitro studies were generally concordant with prior published studies of tumor and obesity models by our group and others.
Data exclusions	In single-cell analyses, outlier populations of β cells with large differences in the number of genes expressed per cell, indicating low quality of the cell populations, were filtered. No other data were excluded.
Replication	Biologic and technical replicates were included as described in the figure legends and Methods. All replicate data presented in the manuscript were concordant.

Randomization	For allocation into experimental groups in treatment studies (e.g., STZ/PBS, JNK inhibitor), mice were randomized following pre-stratification for sex and weight.
Blinding	Blinding was not possible in treatment studies, as physiologic effects were readily apparent (e.g., hyperglycemia with STZ)..

Reporting for specific materials, systems and methods

We require information from authors about some types of materials, experimental systems and methods used in many studies. Here, indicate whether each material, system or method listed is relevant to your study. If you are not sure if a list item applies to your research, read the appropriate section before selecting a response.

Materials & experimental systems

n/a	Involved in the study
☐	☒ Antibodies
☐	☒ Eukaryotic cell lines
☒	☐ Palaeontology and archaeology
☐	☒ Animals and other organisms
☒	☐ Clinical data
☒	☐ Dual use research of concern
☒	☐ Plants

Methods

n/a	Involved in the study
☐	☒ ChIP-seq
☒	☐ Flow cytometry
☒	☐ MRI-based neuroimaging

Antibodies

Antibodies used

Cholecystokinin Rabbit Immunostar 20078 IF/ICC/IHCEM 1:100/1:100/1:1000/1:250
Insulin Rat R&D Systems MAB1417 IF/ICC/EM 1:500/1:500/1:250
Glucagon Rabbit Sigma Aldrich SAB4501137 IF 1:400
Glucagon Mouse Sigma Aldrich G2654 iDISCO 1:2000
Ki67 Rabbit Cell Signaling Technology 9129S IF 1:100
Phospho-c-Jun (Ser73) Rabbit Cell Signaling Technology 3270S WB 1:500
cJun Rabbit Cell Signaling Technology 9165S WB 1:1000
Hsp90 Rabbit Cell Signaling Technology 4877S WB 1:5000
JUN/cJun Rabbit EpiCypher 13-2019 CUT&RUN 0.5 µg
H3K4me3 Rabbit EpiCypher 13-0060 CUT&RUN 0.5 µg
IgG control Rabbit EpiCypher 13-0042 CUT&RUN 0.5 µg
Insulin Guinea Pig Accurate Chemical BMAT5014 IHC 1:300
BiP Rabbit Cell Signaling Technology 3177 IHC 1:400
Synaptophysin Rabbit ThermoFisher Scientific RB-1461 IHC 1:100
Ki67 Rabbit Biocare Medical CRM325 IHC 1:75
Cd45 Rabbit Abcam ab10558 IHC 1:500
Smooth muscle actin (SMA) Mouse ThermoFisher Scientific MS-113 IHC 1:400
Reg2 Goat R&D Systems AF2035 IHC 1:100
Anti-Rabbit IgG (DyLight 800 4X PEG conjugate) Goat Cell Signaling Technology 5151S WB 1:2500
Anti-Rat IgG Alexa Fluor Plus 488 Donkey ThermoFisher Scientific A48269 IF/ICC 1:500
Anti-Rabbit IgG Alexa Fluor Plus 647 Donkey ThermoFisher Scientific A32795 IF/ICC 1:500
Mach2 Rabbit HRP-Polymer Goat Biocare Medical RHRP520 IHC 1:1
Mach2 Mouse HRP-Polymer Goat Biocare Medical MHRP520 IHC 1:1
Peroxidase AffiniPure Anti-Goat Rabbit Jackson ImmunoResearch 305-035-045 IHC 1:500
Peroxidase AffiniPure Anti-Guinea Pig Donkey Jackson ImmunoResearch 706-035-148 IHC 1:500
Biotinylated, Anti-Rabbit Goat Vector Laboratories BA-1000 IHC 1:100

Validation

Antibodies were validated by the manufacturer and their use has been supported by prior publications as listed in their respective datasheets and websites.

Eukaryotic cell lines

Policy information about cell lines and Sex and Gender in Research

Cell line source(s)	Min6 (mouse insulinoma) were obtained from Dr. G. Cline.
Authentication	None of the cell lines used were authenticated.
Mycoplasma contamination	All cell lines tested were negative for Mycoplasma contamination.
Commonly misidentified lines (See ICLAC register)	None used in this study.

Animals and other research organisms

Policy information about studies involving animals; ARRIVE guidelines recommended for reporting animal research, and Sex and Gender in Research

Laboratory animals	Mice (Mus musculus) were used in the study. Mouse strain, genetic background, and age for each experiment are stated in the "Animal studies" section of the Methods and/or figures/figure legends, where applicable.
Wild animals	The study did not involve wild animals.
Reporting on sex	Sex was included as a variable in all analyses and reported in figures, figure legends, and Methods as appropriate.
Field-collected samples	The study did not involve samples collected from the field.
Ethics oversight	Animal studies were approved under the Yale University Institutional Animal Care and Use Committee (IACUC) protocol #20170.

Note that full information on the approval of the study protocol must also be provided in the manuscript.

Plants

Seed stocks	*Report on the source of all seed stocks or other plant material used. If applicable, state the seed stock centre and catalogue number. If plant specimens were collected from the field, describe the collection location, date and sampling procedures.*
Novel plant genotypes	*Describe the methods by which all novel plant genotypes were produced. This includes those generated by transgenic approaches, gene editing, chemical/radiation-based mutagenesis and hybridization. For transgenic lines, describe the transformation method, the number of independent lines analyzed and the generation upon which experiments were performed. For gene-edited lines, describe the editor used, the endogenous sequence targeted for editing, the targeting guide RNA sequence (if applicable) and how the editor was applied.*
Authentication	*Describe any authentication procedures for each seed stock used or novel genotype generated. Describe any experiments used to assess the effect of a mutation and, where applicable, how potential secondary effects (e.g. second site T-DNA insertions, mosiacism, off-target gene editing) were examined.*

ChIP-seq

Data deposition

- [x] Confirm that both raw and final processed data have been deposited in a public database such as GEO.
- [x] Confirm that you have deposited or provided access to graph files (e.g. BED files) for the called peaks.

Data access links<br>*May remain private before publication.*	https://www.ncbi.nlm.nih.gov/geo/query/acc.cgi?acc=GSE280947<br>Reviewer token: ejmrsuswhhmdjgf
Files in database submission	GSM8607874 DMSO treatment (control), IgG antibody (neg control), biological replicate 1<br>GSM8607875 DMSO treatment (control), IgG antibody (neg control), biological replicate 2<br>GSM8607876 DMSO treatment (control), JUN antibody, biological replicate 1<br>GSM8607877 DMSO treatment (control), JUN antibody, biological replicate 2<br>GSM8607878 DMSO treatment (control), anti-H3K4Me3 antibody (pos control), biological replicate 1<br>GSM8607879 DMSO treatment (control), anti-H3K4Me3 antibody (pos control), biological replicate 2<br>GSM8607880 JNK inhibitor treatment (control), IgG antibody (neg control), biological replicate 1<br>GSM8607881 JNK inhibitor treatment (control), IgG antibody (neg control), biological replicate 2<br>GSM8607882 JNK inhibitor treatment (control), JUN antibody, biological replicate 1<br>GSM8607883 JNK inhibitor treatment (control), JUN antibody, biological replicate 2<br>GSM8607884 JNK inhibitor treatment (control), anti-H3K4Me3 antibody (pos control), biological replicate 1<br>GSM8607885 JNK inhibitor treatment (control), anti-H3K4Me3 antibody (pos control), biological replicate 2
Genome browser session<br>(e.g. UCSC)	*Provide a link to an anonymized genome browser session for "Initial submission" and "Revised version" documents only, to enable peer review. Write "no longer applicable" for "Final submission" documents.*

Methodology

Replicates	Two biologic replicates were performed for each sample. There was high agreement between replicates.
Sequencing depth	30 million paired-end 150 bp reads (2x150) per sample.
Antibodies	Cells were processed using the CUTANA ChIC/CUT&RUN Kit (14-1048), JUN/cJun CUTANA CUT&RUN antibody (SKU:13-2019), and positive (anti-H3K4Me3) and negative (IgG) control antibodies from the kit following the manufacturer's protocol.
Peak calling parameters	See "CUT&RUN analysis" section of Methods.
Data quality	See "CUT&RUN analysis" section of Methods.

Software	See "CUT&RUN analysis" section of Methods.
